# Clinical efficacy of a new surgical technique of oral mucosal epithelial transplantation for severe ocular surface disorders

**DOI:** 10.1186/s12886-023-02879-4

**Published:** 2023-04-07

**Authors:** Yuan-Fang Zhu, Wen-Ya Qiu, Ye-Sheng Xu, Yu-Feng Yao

**Affiliations:** 1 Department of Ophthalmology, Sir Run Run Shaw Hospital, Zhejiang University School of Medicine, Hangzhou, China; 2 Key Laboratory for Corneal Diseases Research of Zhejiang Province, NO. 3 East QingChun Rd, Hangzhou, Zhejiang China

**Keywords:** Oral mucosal epithelia, Ocular surface disorder, Limbal stem cell deficiency, Symblepharon

## Abstract

**Background:**

Severe ocular surface disorders are one of the major blinding diseases, and a paucity of original tissue obscures successful reconstruction. We developed a new surgical technique of direct oral mucosal epithelial transplantation (OMET) to reconstruct severely damaged ocular surfaces in 2011. This study elaborates on the clinical efficacy of OMET.

**Methods:**

A retrospective review of patients with severe ocular surface disorders who underwent OMET from 2011 to 2021 at the Department of Ophthalmology, Sir Run Run Shaw Hospital, Zhejiang University School of Medicine was conducted. Patients who were followed up for at least 3 months postoperatively and had sufficient pre or postoperative records were included. Surgical efficacy was evaluated by comparing the best-corrected visual acuity (BCVA), corneal transparency, neovascularization grade, and symblepharon grade. Additionally, postoperative ocular surface impression cytology was used to study the morphology of the newborn epithelial cells.

**Results:**

Forty-eight patients (49 eyes; mean age: 42.55 ± 12.40 years, range:12–66 years) were enrolled in the study. The etiology included chemical burns (30 eyes), thermal burns (16 eyes), explosive injuries (1 eye), Stevens-Johnson syndrome (1 eye), and multiple pterygiums (1 eye). The mean follow-up period was 25.97 ± 22.99 months. Postoperatively, 29 eyes (59.18%) showed improved corneal transparency, 26 eyes (53.06%) had improved BCVA, 47 eyes (95.92%) had a stable epithelium until the final follow-up, 44 eyes (89.80%) had a reduced neovascularization grade. Of the 20 eyes with preoperative symblepharon, 15 (75%) were completely resolved, and five (25%) were partially resolved. Impression cytological studies showed no postoperative conjunctival invasion onto the corneal surface.

**Conclusions:**

OMET is a safe and effective surgical technique for reconstruction in severe ocular surface disorder by maintaining a stable epithelium and reducing the neovascularization and symblepharon grade.

## Introduction

Ocular surface disorders may be caused by a variety of ocular surface diseases and injuries, such as chemical or thermal burns, Stevens-Johnson syndrome (SJS), pemphigoid, infectious diseases, or surgical injuries. In case of severe damage, the corneal surface often undergoes heavy neovascularization (NV), stromal scarring, recurrent epithelial erosion, chronic inflammation [1–4], and combined symblepharon. These changes can limit ocular motility and cause irregular tear fluid dynamics, cicatricial entropion, and hypophasis [5, 6]. Reconstruction procedures for such severe ocular surface disorders are complicated by a lack of original tissues or limbal stem cells required to maintain a healthy ocular surface with clearly separated corneal and conjunctival epithelium.

Different techniques are used for the reconstruction of the limbus, such as auto- and allo- limbal graft transplantation [7–9] and ex-vivo cultivated limbal stem cells [10, 11]. For severe ocular surface disorders, autologous tissue is rarely available and the donor site is at risk of limbal deficiency, while allografts are endangered by a high risk of rejection [12–14]. Cultivated oral mucosal epithelial sheet transplantation (COMET) is an upcoming technique that uses oral mucosal epithelial stem cells as a substitute for corneal limbal stem cells with good efficacy in clinical use [15–17]. Nevertheless, the complexities associated with cultivation techniques limit the possibility of their widespread use.

To mitigate these logistic limitations with COMET, direct transplantation of an oral mucosal graft, including circumferentially-trephined graft transplantation [18, 19] and simple oral mucosal epithelial transplantation (SOMET) [20, 21], were performed with good results. We modified the direct oral mucosal transplantation technique for treating patients with severe ocular surface disorders since 2011. This study was then designed to analyze the clinical efficacy of this modified surgery technique in ocular surface reconstruction.

## Methods

### Ethical compliance

This retrospective study was approved by the Ethics Committee of Sir Run Run Shaw Hospital (approval number: 20200716-266), Hangzhou, China. All procedures involving human participants were performed in accordance with the Declaration of Helsinki.

### Patients

We retrospectively reviewed patients with a severe ocular surface disorder who had undergone an OMET surgery from 2011 to 2021 and were followed up for at least 3 months after surgery. Limbal stem cell deficiency (LSCD) in this study was classified according to the global consensus statement for staging LSCD published by the Corneal Society in 2019 [22]. Patients with stage III LSCD with or without symblepharon, or stage IC and IIB LSCD combined with symblepharon were designated as severe ocular surface disorders. Patients with stage IC and IIB, whose limbus had some remained function, were classified into partial LSCD group. While patients with stage III, who suffered a total loss of limbal function, were classified into total LSCD group. We extracted patient data from the medical records and patients with insufficient pre or postoperative records were excluded from the study.

Prior to the surgery, 0.1% fluorometholone (Santen, Osaka, Japan) and 0.5% levofloxacin (Cravit; Santen, Osaka, Japan) were given four times a day for two weeks to minimize inflammation and prevent bacterial infection [23]. Additionally, compound chlorhexidine gargles (1.2 mg/ml chlorhexidine and 0.2 mg/ml metronidazole) were used twice a day for three days to improve oral hygiene.

### Surgical procedures of OMET

**Fig. 1:**
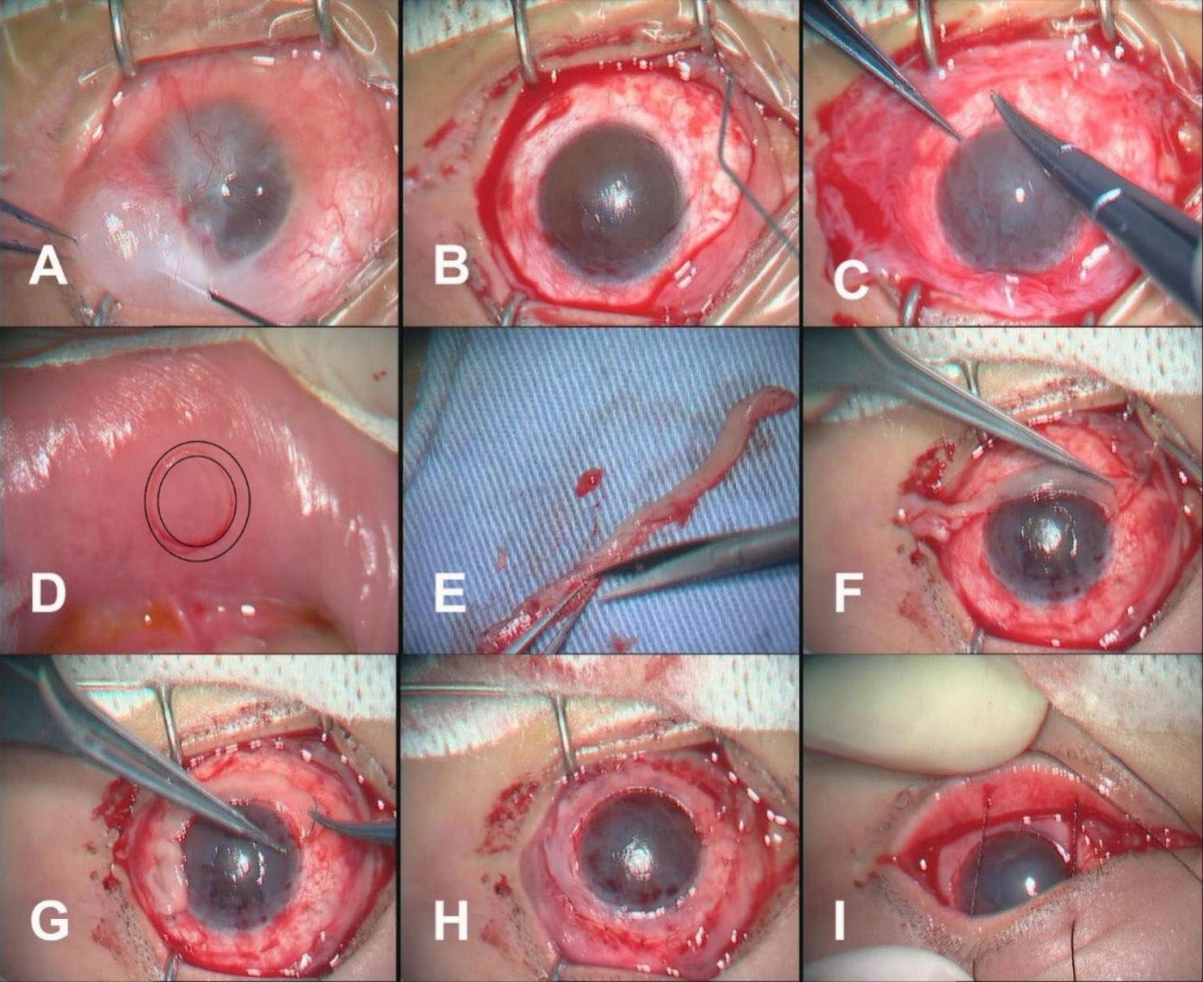
The surgical procedures of OMET. A: Subconjunctival injection of saline. B: Removal of the fibrovascular tissue to expose the sclera and corneal surface. C: Placement of the cryo-AM and suturing. D: Harvesting of the oral mucosa graft from the lower lip. A double-ring incision (black circle) was made, and the mucosa between the two circles was harvested. E: Shaving off the subepithelial tissue. F and G: Suturing the graft onto the cryo-AM in the limbus area. H: Suturing the other side of the graft to the conjunctiva. I: Temporary suturing of the eyelids at the end of the surgery. OMET, oral mucosal epithelial transplantation; cryo-AM, cryopreserved amniotic membrane

All surgeries were performed by a single surgeon (YF. Y). The procedure was carried out under general anesthesia; the endotracheal tube was secured to one side of the mouth to allow for harvesting the oral mucosal graft (described below). Normal saline was injected subconjunctivally to detach the epithelium and the fibroblastic connective tissue (Fig. 1A). The extent of the peritomy was determined by the degree of LSCD. Subconjunctival fibrovascular tissue was extensively removed to expose the sclera and corneal stroma. Symblepharons, if combined, were released. The conjunctival epithelium was retained as much as possible, especially in eyes with severe symblepharon (Fig. 1B).

The debrided ocular surface was then coated with a smooth layer of cryopreserved amniotic membrane (cryo-AM) as a basement membrane, which was extended to the deep fornix area, including the corneal and scleral surfaces.

In all cases, the cryo-AM was sewn along the limbus in a circle and then using an interruptedly sutured in the fornix area, using a 10 − 0 nylon suture (Fig. 1C). The lower lip was sterilized again with povidone-iodine. A 1.5-2 mm-wide oral mucosa graft was harvested from the lower lip (next to the gum) (Fig. 1D), and the subepithelial tissue was shaved off as much as possible (Fig. 1E). The size of the graft was determined by the stage of LSCD. The oral mucosal epithelial graft was washed with gentamicin sulfate (0.16 mg/ml) three times, followed by normal saline three times; next, interrupted sutures were applied at the limbal area above the cryo-AM (Fig. 1F-H). Finally, the middle of the eyelids by was secured by interrupted sutures using a tarsorrhaphy wire (Fig. 1I). In the eyes with grade III and grade IV symblepharon (n = 3), OMET was combined with autologous conjunctival transplantation.

### Postoperative management

To mitigate inflammation and prevent bacterial infection, all patients were given oral prednisolone (0.5 mg/kg/day) and levofloxacin tablets (0.5 g /day) for three days postoperatively. Topical eye drops, including 0.1% fluorometholone (Santen, Osaka, Japan), 0.5% levofloxacin (Cravit; Santen, Osaka, Japan) and preservative-free 0.1% sodium hyaluronate (Hycosan, EUSAN, Saarbrücken, Germany) were used together throughout the recovery period. Until the epithelium was restored, eye drops were applied four times a day. After the epithelium was complete, the frequency of eye drops was gradually tapered down to thrice, twice, and once a day, and eventually stop. Additionally, the compound chlorhexidine gargles were used twice a day for three days postoperatively. Both the oral and eyelid sutures were removed in the second week (10–12 days after surgery). Bandage contact lenses were used in patients with suitable conjunctival sac capacity before epithelization completed. Patients were asked to follow-up regularly.

Once epithelization was completed and stable, impression cytology was performed routinely in all patients to study the morphology of the new epithelial cells growing on the ocular surface. We followed Tseng’s method [24] for impression cytology, using cellulose acetate filter paper (Millipore filter paper, US; pore size: 0.22 μm) and Periodic acid Schiff (PAS) staining photographed under a light microscope.

Additionally, epithelial cell samples were taken from all patients as controls – oral epithelial cells, and normal corneal and conjunctival epithelial cell samples from the healthy eye of patients with unilateral disease.

### Evaluation of efficacy

**Fig. 2:**
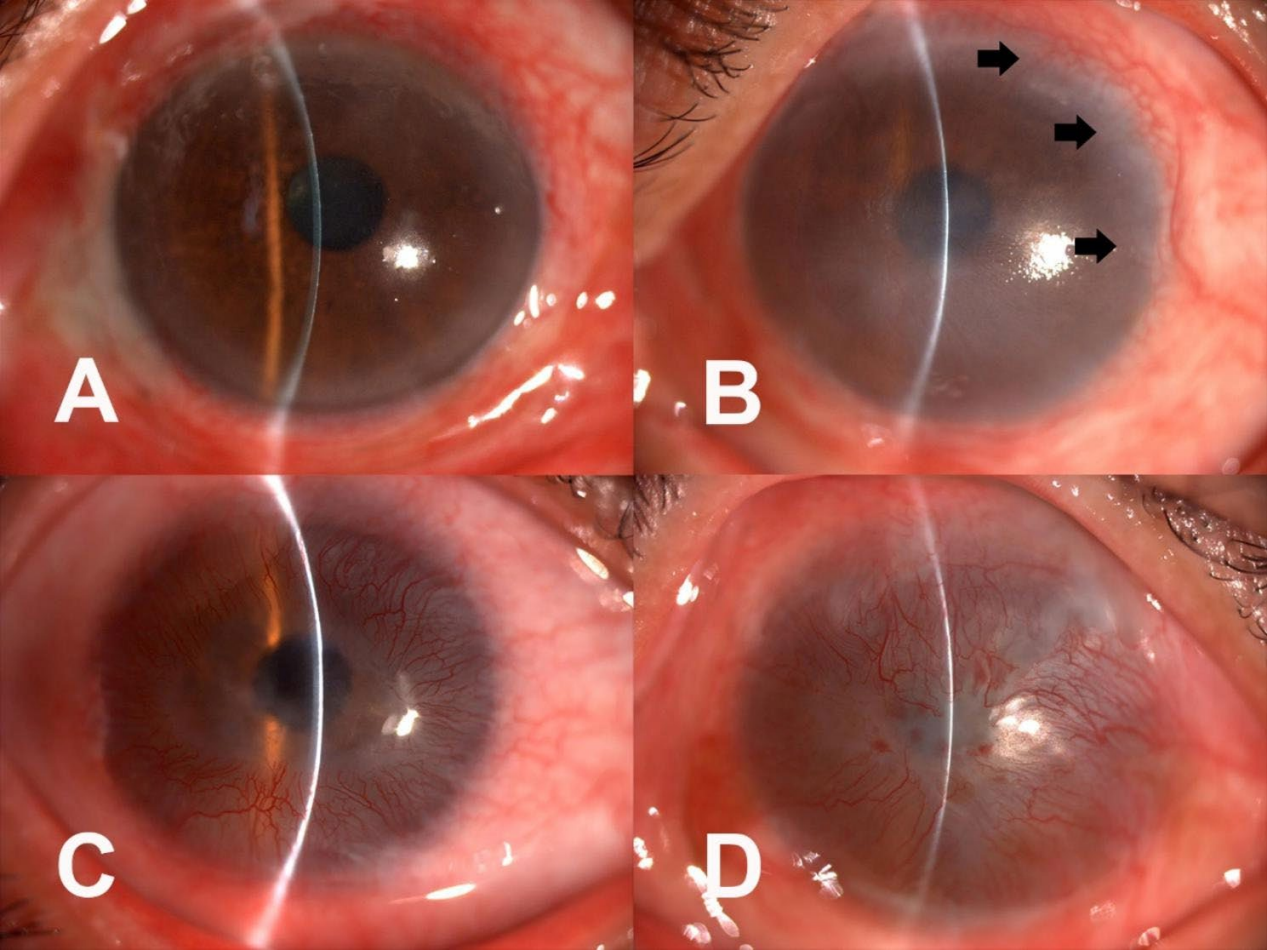
Grading neovascularization in the eyes. A: Grade 0, no invasion of the cornea; B: Grade 1, peripheral invasion of the cornea (black arrows), 1 to 2 mm inside the limbus; C: Grade 2, mid-peripheral invasion; D: Grade 3, invasion of the entire cornea

**Fig. 3:**
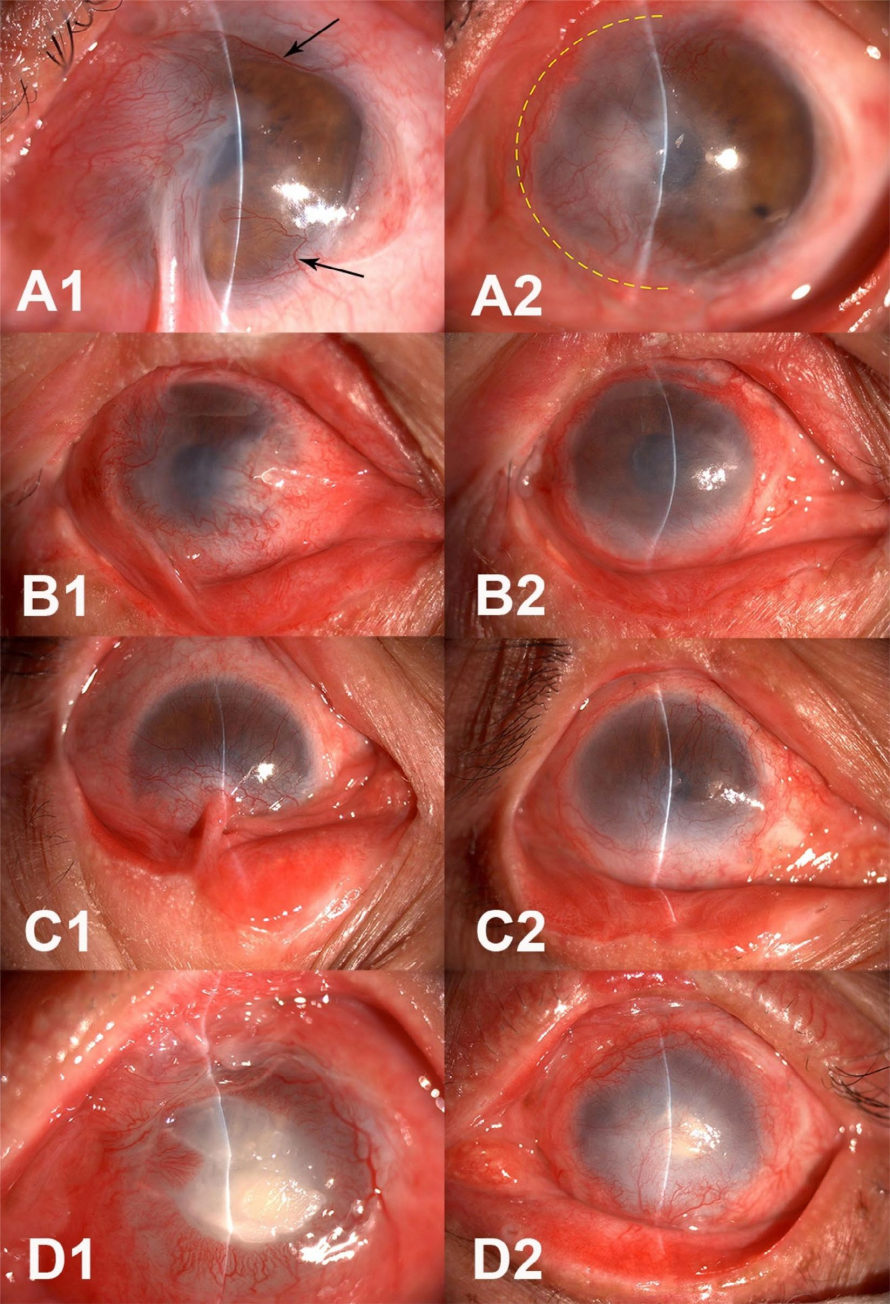
Pre- and postoperative slit-lamp photographs for four patients with symblepharon. A1, B1, C1, and D1 are preoperative photographs showing grades I, II, III, and IV symblepharon, respectively. A2, B2, C2, and D2 were taken 14 months, 8 months, 3 months, and 12 months, respectively, after surgery for patients A, B, C, and D. A2 has no symblepharon, B2 and C2 show grade I symblepharon, and D2 shows grade II symblepharon Patient A was a 56-year-old man, whose left eye was damaged by chemical burn. The oral mucosa graft was transplanted at the nasal half part of limbus as the yellow line shown in A2. The neovascularization (NV) in the non-operated area (A1, black arrows) were no longer seen after surgery (A2)

The clinical efficacy of the surgery was evaluated by comparing the following pre- and postoperative factors:

1. Best-Corrected Visual Acuity (BCVA): BCVA was evaluated by the standard logarithmic visual acuity chart. Visual acuity lower than 1/100 (2 logMAR) would be evaluated by counting fingers (CF), hand motion (HM), and light perception (LP). Postoperative BCVA was recorded as the result of the final follow-up visit.
2. Corneal transparency: Pre and postoperative corneal transparency was recorded by slit-lamp photographs and compared.
3. Epithelization: Complete and stable epithelization was defined as the patient having no epithelial defects on the cornea or the conjunctival surface and remaining so throughout the follow-up period. In patients with recurrent epithelial defects, the epithelization time was recorded as the time when epithelization was complete and no further defects occurred. Those with persistent epithelial defects and incomplete epithelization even after three months of follow-up were recorded as treatment failures. Weather age, etiology, preoperative surgical history, preoperative PEDs and the LSCD grade were related to the postoperative epithelization time were analyzed.
4. NV grading: Ocular surface NV was graded by an experienced ophthalmologist using the slit-lamp photography results based on the method described by Satake Y, et al. [16]. The ophthalmologist was blinded to the patient’s characteristics. The following grades were awarded according to the extent of the NV: grade 0, no invasion of the cornea; grade 1, peripheral invasion of the cornea 1–2 mm inside the limbus; grade 2, mid-peripheral invasion, greater than grade 1 but not involving the pupillary area; and grade 3, invasion of entire cornea, extending to the pupillary area (Fig. 2).

5. Symblepharon grading: The severity of symblepharon was graded based on the length of the remaining conjunctiva, including the depths of the tarsal and bulbar conjunctiva. Grades I was assigned if the remaining conjunctiva was equal to or longer than the length of the normal palpebral conjunctiva in that area, grade II if it was shorter than the normal palpebral conjunctiva but equal to or longer than the normal tarsus in that area, grade III if it was shorter than the normal tarsus, and grade IV if it was close to zero (ankyloblepharon) (Fig. 3) [5].

### Statistical analysis

All data analyses were performed using SPSS (IBM SPSS Statistics for Windows, Version 23.0. Armonk, NY: IBM Corp). A test of normality of distribution was done for all continuous variables; accordingly, we used Student’s t-test to compare the normally distributed variables and the Wilcoxon rank-sum test to analyze the non-normally distributed data. The Wilcoxon signed-rank test was used to compare ordinal categorical variables and their correlations were assessed using Spearman’s rank correlation analysis. A p-value of < 0.05 (two-sided) was considered statistically significant.

## Results

A total of 48 (45 males, 3 females) patients with a mean age of 42.55 ± 12.40 years (range: 12–66 years) were enrolled in the study. This patient cohort included 49 injured eyes with multiple etiologies including chemical burns (30 eyes), thermal burns (16 eyes), explosive injuries (1 eye), SJS (1 eye), and multiple pterygiums (1 eye). Twenty-eight patients had bilaterally injuries, and 20 patients had unilateral injuries. A diagnosis of LSCD was confirmed by impression cytology that revealed corneal surface invasion by conjunctival epithelial cells and goblet cells [25–27]. Further, 33 eyes were categorized as stage III LSCD, 14 eyes with stage IIB, and two eyes with stage IC. Besides LSCD, 20 eyes had different degrees of symblepharon, nine had persistent epithelial defects (PEDs), and 25 had undergone a previous ocular surface reconstruction surgery without OMET (Table 1).

**Table 1:** Preoperative ocular surface status

Variables	Number	LSCD Stage			Ocular surface surgery history	PEDs	Symblepharon
		IC	IIB	III			
Chemical burns	30		4	26	12 AMT (n = 7), PKP (n = 2), LK (n = 1), KLAL (n = 2)	7	11
Thermal burns	16	2	9	5	11 AMT (n = 8), LK (n = 2), Deb (n = 1)	1	7
Explosion	1			1	1 (PKP)		1
SJS	1			1	No	1	
MP	1		1		1 (Deb)		1
Total	49	2	14	33	25	9	20

LSCD, limbal stem cell deficiency; PEDs, persistent epithelial defects; AMT, amniotic membrane transplantation; PKP, penetrating keratoplasty; LK, lamellar keratoplasty; KLAL, kerato-limbal allograft; Deb, debridement of corneal surface invasion tissue; SJS, Stevens-Johnson syndrome; MP, multiple pterygiums.

The average postoperative follow-up period was 25.97 ± 22.97 months. One-year follow-up data were available for 32 eyes (65.31%), and 2-year follow-up data were available for 22 of these eyes.

**Fig. 4:**
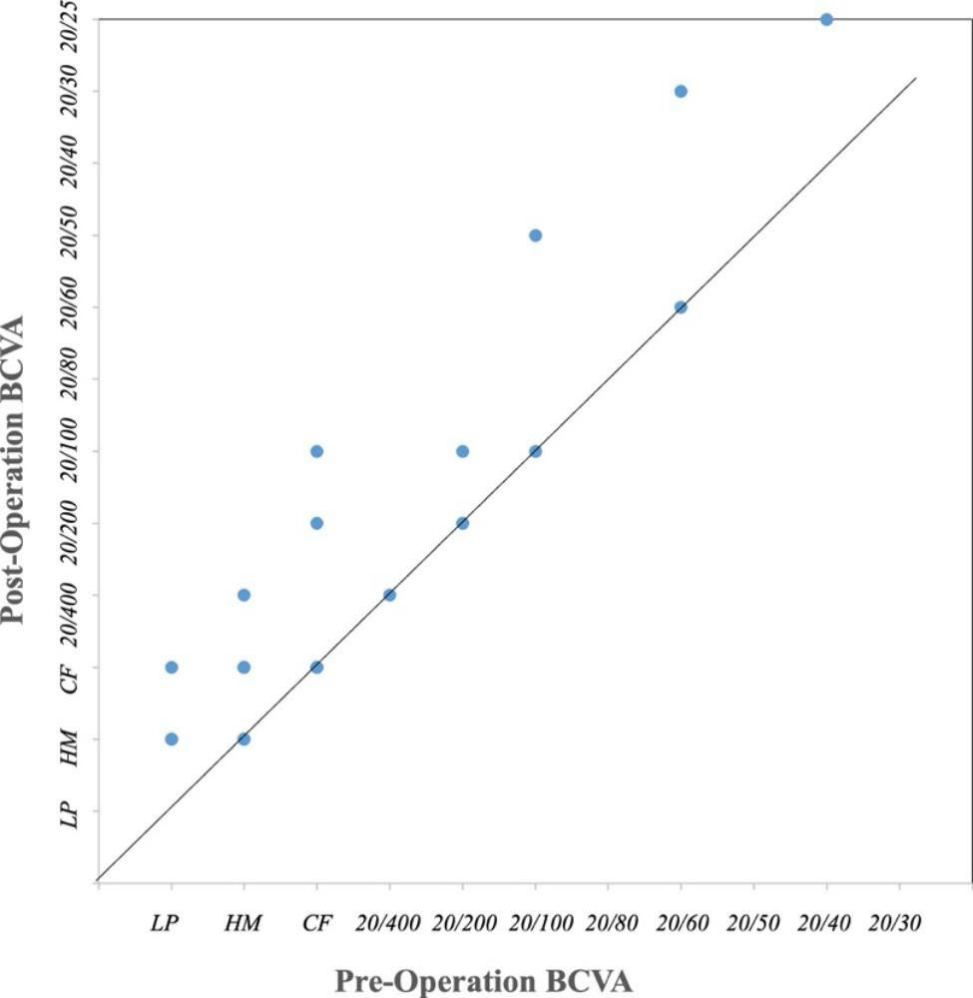
BCVA measured before and after surgery (at the last follow-up). Twenty-six cases (53.06%) showed improved BCVA after surgery and the rest remaining showed no change. BCVA, best-corrected visual acuity; LP, light perception; HM, hand movement; CF, count finger

After the fibroconnective tissue excision and corneal surface re-epithelization, 26 cases (53.06%) showed improved BCVA (Fig. 4), and 29 eyes (59.18%) had better corneal transparency than before. Forty-seven eyes (95.92%) showed complete epithelization within a mean time of 26.04 ± 26.43 days; only two eyes failed and remained PED at the last visit but without stromatolysis.

Further, we found that the postoperative epithelization time was related to the LSCD grade and preoperative PEDs (R = 0.52 and R = 0.39, respectively; p < 0.01). Epithelization was faster in patients with partial LSCD than in patients with total LSCD (12.19 ± 5.90 days versus 32.80 ± 30.41 days, respectively; p < 0.05). Patients with preoperative PEDs took longer than those without PEDs to achieve complete epithelialization (53.71 ± 34.35 days versus 21.20 ± 21.93 days, respectively; p < 0.05). While age, etiology and preoperative surgical history had no relation to the epithelization time.

**Fig. 5:**
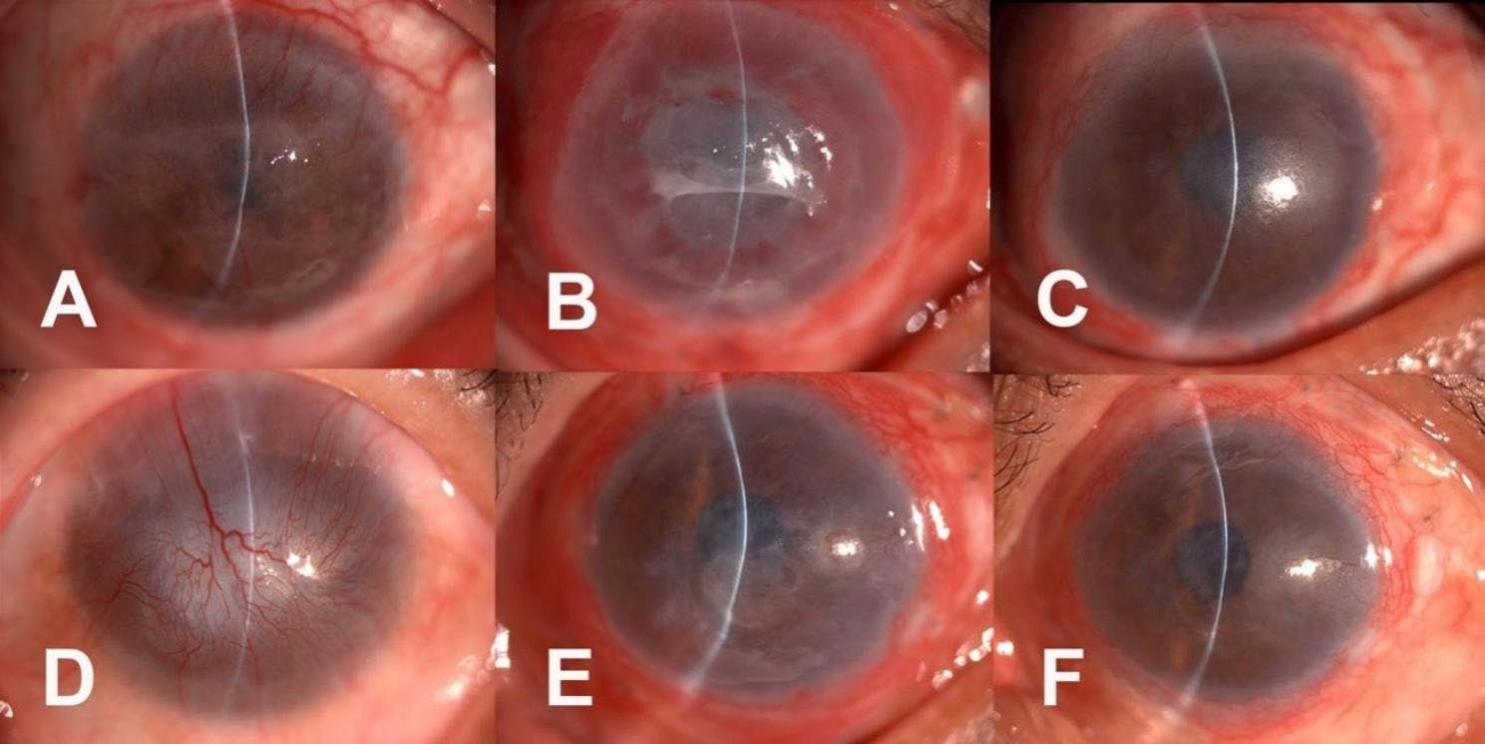
Ocular surface changes after surgery A-C: In a 26-year-old male patient diagnosed with SJS. A: Preoperative ocular surface status. B: One month after OMET, the corneal surface epithelization had not been completed, and intensive tiny NV occurred. C: Seven months after OMET, corneal surface epithelization was completed, and the NV was diminished D-F: In a 40-year-old male patient sustaining a chemical burn. D: Preoperative ocular surface status. C: One month after OMET, the corneal surface epithelization had not been completed yet F: Six months after OMET, epithelization had been completed and the patient had a better cornea transparency compared to picture E SJS, Stevens-Johnson syndrome; OMET, oral mucosal epithelial transplantation; NV: neovascularization

**Fig. 6:**
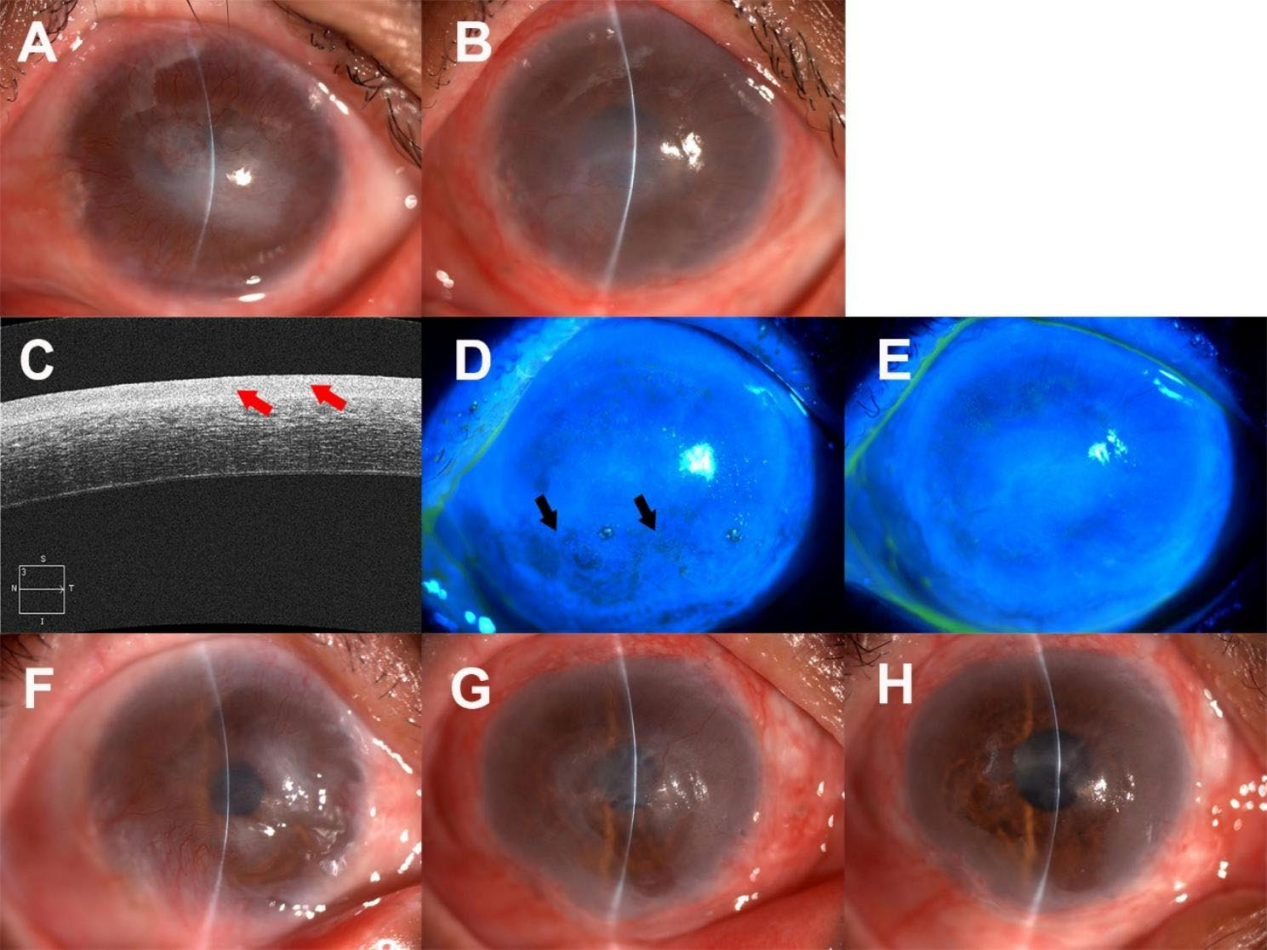
Features of the postoperative corneal surface epithelia A-E: From a 48-year-old female patient who sustained thermal burns. A: Preoperative ocular surface status. B: At 45 days after the OMET, the corneal surface epithelization had completed and less NV compared to preoperative status. C: High-resolution OCT (Zeiss, Oberkochen, Germany) examination for the cornea at 45 days after OMET showed a thick epithelial layer (red arrows). D: Fluorescein staining of tear fluid showed a complete and smooth corneal epithelium, the dark stained (black arrows) part represents clusters of mucins adhering on the corneal surface. E: After washing with artificial tear drops, the mucins in picture D are no longer seen F-H: From a 57-year-old male patient who sustained a chemical burn. F: Preoperative ocular surface status. G: Three months after OMET, showing a semi-transparent corneal epithelium. H: Thirteen months after OMET, the corneal epithelium becomes thinner and more transparent than at three months

Postoperatively, patients who were followed up for more than one year showed greater corneal NV before completing corneal epithelization and decreased corneal NV with increased corneal transparency after completing epithelization (Fig. 5). The new corneal surface epithelia were less transparent than normal corneal epithelia. When stained with fluorescein, the corneal surface was smooth, with abundant clusters of mucins that could be washed away by artificial tears. One-year after the surgery, the epithelia were still transforming to become more transparent than in earlier postoperative stages. (Fig. 6).

**Fig. 7:**
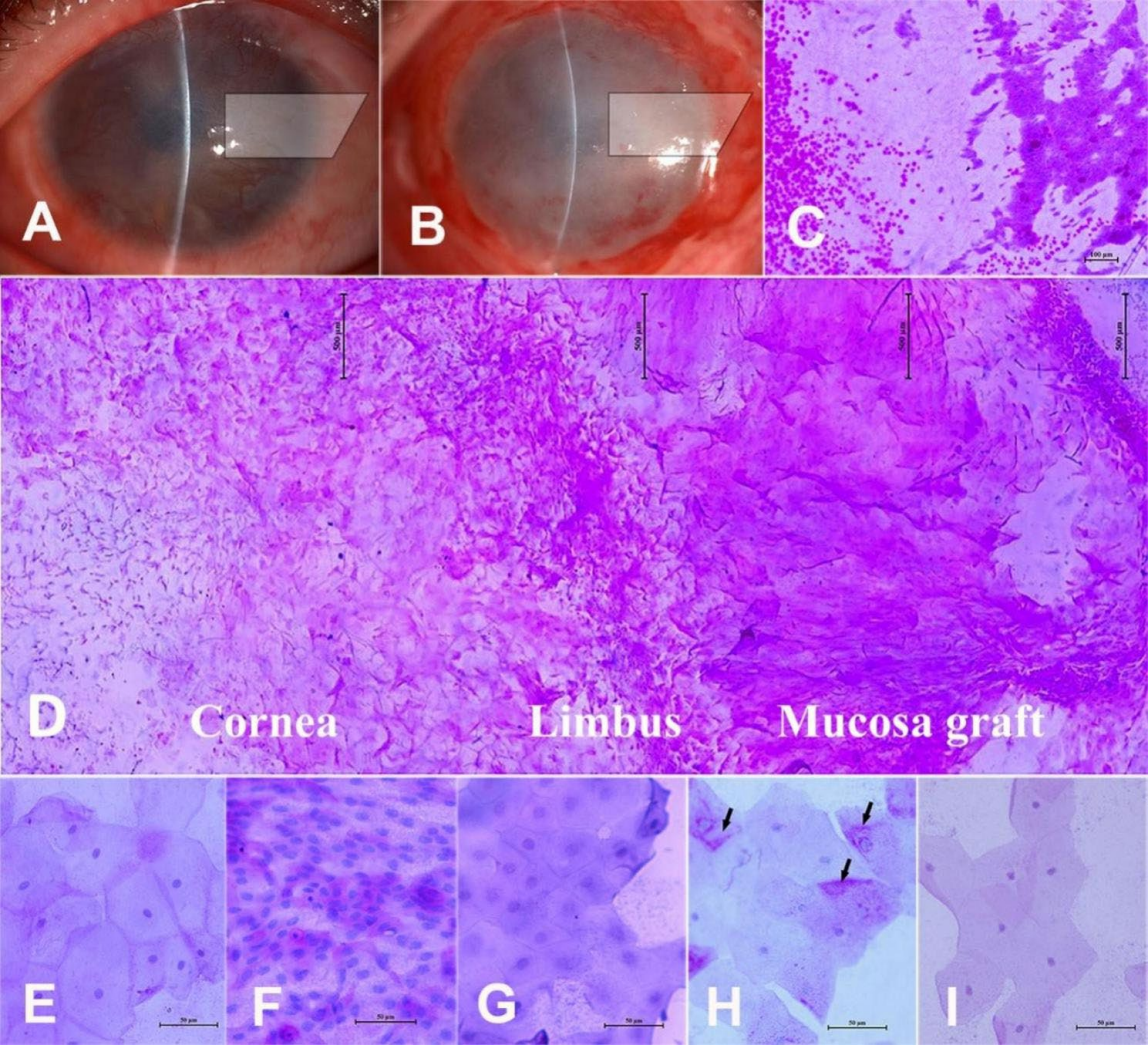
IC studies for corneal epithelium after OMET. A, B: Slit-lamp photographs. C-I: PAS staining for corneal surface epithelium, photographed under light microscope A-D: From a 34-year-old male patient who sustained chemical burns. chemical burn. A: Preoperative status of the ocular surface. The trapezoid shows the filtering paper sample position for IC. B: Twelve days after surgery, the trapezoid shows that the IC examination was taken at the same position as the preoperative site. C: IC results for A, showing conjunctival epithelia cell features. D: IC result for B, 12 days after OMET. Cells at the mucosal graft position, at the limbus and the cornea surface position were morphologically different E-G: IC control pictures. E: Normal oral epithelia cells, taken from the patient himself. F: Normal conjunctival epithelial cells. G: Normal corneal epithelia cells. F and G were taken from the healthy eye of a 30-year-old male patient with unilateral chemical injury H-I: IC results for the eye in Fig. 6H. H: Showing epithelial cells in the peripheral corneal area, next to the limbus. There were a lot of PAS-positive substances in the cells (black arrows). I: Showing cells in the central corneal area, next to the pupil, where the epithelium was nearly transparent. There were fewer PAS-positive substances in cells Scale bars: C, 100 μm; D, 500 μm; E-I, 50 μm IC, impression cytology; OMET, oral mucosal epithelial transplantation; PAS, Periodic acid–Schiff

Impression cytology was performed in 47 patients (47 eyes) whose surface epithelium was complete and stable. Among them, 29 eyes (59.18%) showed complete epithelialization within two weeks after surgery, hence, we performed impression cytology on these eyes on days 12–14 after the surgery. The impression cytological study area covered the whole mucosa graft and a 3–4 mm corneal area. We observed that the newly transdifferentiated epithelial cells were morphologically different from the corneal or conjunctival epithelial cells and more like the oral mucosal epithelial cells of their origin (Fig. 7). Cells present near the graft area were small, with a high nucleus-to- cytoplasm ratio. They were irregular in shape, had multiple layers, and were darkly stained by PAS staining. When migration onto the central corneal area, they increased in size and became more regular in shape, with a reduced nucleus-to-cytoplasm ratio, they were more lightly stained and had fewer layers. No conjunctival or goblet cells were detected on the corneal surface in the examined cases.

The degree of postoperative NV decreased in 44 eyes (89.80%). Twelve eyes (24.49%) showed grade 0 NV, 27 eyes  (55.10%) had grade 1, nine eyes (18.37%) had grade 2, and only one eye (2.04%) had grade 3 NV after OMET. These results were in sharp contrast with the preoperative NV assessment results: no eyes with grade 0 NV, two eyes (4.08%) with grade 1, 10 eyes (20.41%) with grade 2, and 37 eyes (75.51%) with grade 3. The remaining five eyes had the same neovascularization invasion grade as their preoperative value (Table 2). This difference between pre and postoperative NV grading was statistically significant (p < 0.01).

Twenty eyes had symblepharon before surgery – 13 eyes (65%) with grade I, four (20%) with grade II, one (5%) with grade III, and two (10%) with grade IV. After surgery, 15 eyes (75%) were completely released, and five (25%) were partially released (two changed from grade II to grade I, one changed from grade III to grade I, and two changed from grade IV to grade II) (Table 3). This difference between pre- and postoperative symblepharon was statistically significant (p < 0.01).

**Table 2:** The extent of neovascularization before and after surgery

Neovascularization grade	Preoperatively (Number of eyes)	Postoperatively (Number of eyes)
**Grade 0**	0	12
**Grade 1**	2	27
**Grade 2**	10	9
**Grade 3**	37	1

**Table 3:** Symblepharon grading before and after surgery

Symblepharon grade	Preoperatively (Number of eyes)	Postoperatively (Number of eyes)
**Grade 0**	0	15
**Grade I**	13	3
**Grade II**	4	2
**Grade III**	1	0
**Grade IV**	2	0

No complications or adverse events were reported during the operations. Postoperative follow up showed that the oral mucosal epithelial grafts survived well in all patients, and no rejection reaction was observed. Eighteen eyes developed a mild hematocele under the amniotic membrane but recovered in 10–45 days without any sequelae. The donor sites in the lower lips healed in 9–12 days. Only one patient complained of stabbing pain during the first month; no other complications were observed during the whole follow-up period.

## Discussion

Currently, the management strategies for limbal reconstruction include autologous or allogenic limbal stem cell transplantation [7, 8], ex-vivo expansion of limbal epithelial stem cells [10, 11], and using alternative sources of epithelial cells transplantation [15–17, 28–31]. Autologous tissue is limited in the case of a severely damaged limbus; also, complications, such as conjunctival encroachment, graft dislodging, and progressive conjunctivalization have been reported during the postoperative period [13]. On the other hand, allogenic limbal stem cell transplantation is often accompanied by immune reactions, and the use of systemic immunosuppression is hindered by its own adverse effects [12, 32, 33]. Alternative stem cell sources include oral mucosal epithelial cells [15, 16], mesenchymal stem cells [28, 29], hair follicle stem cells [30], and human pluripotent stem cells [31]. Among them, COMET is a relatively frequently used modality that is reported to be effective [15–17, 34–36]; other aforementioned strategies are seldom applied to clinical use and lack scientific evaluations. However, due to the requirement for dedicated techniques and experimental equipment, COMET has not been used commercially, especially in underdeveloped areas where ocular surface disorders are common and caused by chemical or thermal burns [37].

Direct transplantation of oral mucosal sheets was first performed by Liu J who reported good results [18]. Although there were instance of recurrent NV and postoperative epithelium was opaquer than the normal corneal epithelium, the study results verified that direct oral mucosal transplantation was useful in limbal reconstruction. SOMET is also a direct transplantation of oral mucosa, based on the principle of simple limbal epithelial transplantation (SLET), and can successfully regenerate the epithelial layer in complicated ocular surface reconstruction [20]. Compared to COMET, SOMET techniques have greater advantages in terms of surgical skills, an abundance of donor graft tissue, procedural safety, and ease of harvesting.

In this study, we used OMET, which is a modification of direct oral mucosal transplantation. We took the whole epithelial layer of the oral mucosa (including the stem cell layer and some subepithelial tissue) as a graft and transplanted it onto the AM in the LSCD area. The whole of the conjunctival epithelium was retained and sutured to the outer side of the oral graft; the inner side of the oral graft remained free. Once the graft survived, new epithelial cells were generated by the stem cells which spread continuously over the cornea until the whole surface was epithelized. Acting as the basement membrane, the AM provided a smooth and firm surface for the epithelial cells to grow on and migrate across from the limbus area to the center of the cornea surface. Unlike Liu’s surgery, the oral graft we harvested was thinner, and postoperative expansion of the stem cells was single-sided (only to the corneal surface). Compared to SOMET, a larger harvest site wound in the mouth was a shortcoming of OMET; however, there were no further complications in the lower lips during the long-term follow-ups. Also, transplantation of the whole graft tissue helped minimize the rate of graft loss that happened in SOMET; there was no graft loss in our study. Another apparent benefit of OMET was the barrier function; based on the impression cytological results, there was no evidence of postoperative conjunctival invasion which was seen in SOMET. Although NV was recurrent in many cases, the postoperative NV picture in OMET was quite different. There were large, looped vessels in the oral graft area; smaller blood vessels branched out vertically from the loop and extended onto the corneal surface with the new epithelium.

However, OMET was less efficient in restoring visual function; only 26 eyes (53.06%) showed improvement in BCVA. Although the corneal scar was one of the contributing factors, another possible reason was that the epithelial cells generated from the graft were different from normal corneal epithelial cells. According to the impression cytological results, the new cells were more like the oral mucosal epithelial cells, and some of the epithelial cells showed a somewhat transparent outlook, while some remained opaque (Figs. 6H and 7 H, and 7I). The governing mechanism remains unknown. An animal study showed that after two weeks of OMET, a K3 positive and K13 negative stratified epithelium was found covering the corneal surface [38]. Whether in-vivo transplantation would stimulate the stem cells of the oral epithelial graft to transform in a human was unknown, which is also a limitation of the present study. To address this, the identification of specific cell markers and characteristics of the new epithelial cells and their functions needs to be studied.

In this study, analysis of the epithelialization time suggested that the existence of preoperative PEDs was closely related to recovery time, i.e., the postoperative epithelialization time was longer in eyes with PED. The two eyes with postoperative PEDs in this study had recurrent epithelial defects for years before the reconstruction surgery, which may have been caused by the lid margin keratinization, entropion, and trichiasis. A PED may indicate an underlying inflammatory process and poor ability of the ocular surface to self-heal, and correction of these lid margin pathologies before OMET may offer better results. Therefore, it can be reasonably assumed that a healthier preoperative ocular surface status allows for a shorter postoperative epithelialization time. This assumption was further supported by the fact that the group of patients with partial LSCD had a shorter epithelialization time than those with total LSCD (12.19 ± 5.90 days versus 32.80 ± 30.41 days). Another interesting observation in patients with partial LSCD was that the NV around the corneal limbus in the non-operated areas also diminished after surgery (Fig. 3A1–2). A recession of these vessels might have been due to the healthier and more stable surface environment which developed after the OMET, with less inflammation and a completed epithelium.

Another encouraging result of the study was that OMET was effective in patients with SJS. Patients with SJS often have severe vision loss and discomfort due to persistent inflammation and NV of the ocular surface, and there is no effective management other than corticosteroids and immunosuppressors [39, 40]. COMET allows successful and sustained restoration of ocular surface anatomy with significant functional improvements [41]. In our study, although the epithelialization took over 2 months and was accompanied by intensive NV in the SJS patient, in the later postoperative stages, the corneal surface became smooth with less vascular invasion (Fig. 5B–C). However, there was only one SJS patient in this study with insufficient follow-up time (7 months), so the result may not be entirely representative.

For patients with unilateral disease, admittedly, both conjunctival limbal autograft (CLAU) and SLET would achieve remarkable results [4, 9, 23]. In such cases, OMET may be an alternative procedure to save the donor cells in the better eye; however, because the newborn epithelial cells after OMET are not as transparent as normal corneal epithelia, and the ingrowth of new vessels could not break off thoroughly, subsequent corneal transplantation is not suitable in such eyes.

A limitation of this study is that the method we used to diagnose LSCD was conventional impression cytology which might be insufficient to confirm the diagnosis in difficult cases. Other latest techniques, such as in-vivo confocal microscopy and impression cytology with immunofluorescence staining [35, 42], would provide stronger evidence, especially when there is no correlation between clinical manifestation (NV) and conventional impression cytology. Another weakness of this study is the difference in follow-up time between patients. The longest follow-up time was over 90 months for two eyes whose ocular surface remained smooth and without any complications, while the shortest follow-up time was 3 months for three eyes. For more systematic and reliable results, a longer and more consistent follow-up period is required for all cases.

To summarize, this study demonstrated that OMET surgery could be a viable option for severe ocular surface disorders requiring limbal reconstruction to achieve a stable ocular surface with decreased NV and symblepharon grading. The surgical technique is easy to learn and can be safely used.

## Data Availability

The datasets generated and/or analyzed during the current study are not publicly available because we are not able to permit any possibility of identifying persons from treatment history regardless of data anonymity, but data are available from the corresponding author on reasonable request.

## Abbreviations

OMET: Oral Mucosal Epithelial Transplantation
COMET: Cultivated Oral Mucosal Epithelial Sheet Transplantation
SOMET: Simple Oral Mucosal Epithelial Transplantation
LSCD: Limbal Stem Cell Deficiency
PED: Persistent Epithelial Defects
BCVA: Best-corrected Visual Acuity
NV: Neovascularization
cryo-AM: Cryopreserved Amniotic Membrane
CLAU: Conjunctival Limbal Autograft
SLET: Simple Limbal Epithelial Transplantation
